# Cordycepin mitigates spermatogenic and redox related expression in H_2_O_2_-exposed Leydig cells and regulates testicular oxidative apoptotic signalling in aged rats

**DOI:** 10.1080/13880209.2022.2033275

**Published:** 2022-02-17

**Authors:** Spandana Rajendra Kopalli, Kyu-Min Cha, Jae Youl Cho, Si-Kwan Kim, Sushruta Koppula

**Affiliations:** a Department of Bioscience and Biotechnology, Sejong University, Seoul, Republic of Korea; b Department of Integrated Biosciences, College of Biomedical & Health Science, Konkuk University, Chungju, Republic of Korea; c Business Incubator Center 406, D&L Biochem, Chungju-Si, Republic of Korea; d Department of Integrative Biotechnology, Sungkyunkwan University, Suwon, Republic of Korea

**Keywords:** Oxidative stress, lipid peroxidation, testosterone, apoptosis, catalase, glutathione, spermatogenic factors

## Abstract

**Context:**

Cordycepin (COR), from *Cordyceps militaris* L., (Cordycipitaceae), is a valuable agent with immense health benefits.

**Objective:**

The protective effects of COR in ageing-associated oxidative and apoptosis events *in vivo* and hydrogen peroxide (H_2_O_2_)-exposed spermatogenesis gene alterations in TM3 Leydig cells was investigated.

**Materials and methods:**

Male Sprague-Dawley rats were divided into young control (YC), aged control (AC) and COR treated (COR-20) aged groups. COR-20 group received daily doses of COR (20 mg/kg) for 6 months. Cell viability and hormone levels were analysed by MTT [3-(4,5-dimethylthiazol-2-yl)-2,5-diphenyltetrazolium bromide] and enzyme immunoassay kits with COR treated at 1, 5, and 10 μg/mL. Oxidative enzymes, spermatogenic, and apoptotic expression in testis tissues were evaluated by Western blotting and real-time RT-PCR.

**Results:**

COR treatment (1, 5, and 10 μg/mL) significantly (*p <* 0.05 ∼ *p <* 0.001) inhibited the H_2_O_2_-induced decrease in the percentage of viable cells (from 63.27% to 71.25%, 85.67% and 93.97%, respectively), and reduced the malondialdehyde (MDA) content (from 4.28 to 3.98, 3.14 and 1.78 nM MDA/mg protein, respectively). Further, the decreased antioxidant enzymes (glutathione-*S*-transferase mu5, glutathione peroxidase 4 and peroxiredoxin 3), spermatogenesis-related factors (nectin-2 and inhibin-α) and testosterone levels in H_2_O_2_-exposed TM3 cells were significantly (*p <* 0.05 ∼ *p <* 0.001) ameliorated by COR. In aged rats, COR (20 mg/kg) restored the altered enzymatic and non-enzymatic antioxidative status and attenuated the apoptotic p53 and Bax/Bcl-2 expression significantly (*p <* 0.05).

**Conclusion:**

COR might be developed as a potential agent against ageing-associated and oxidative stress-induced male infertility.

## Introduction

One of the leading causes of male infertility is the susceptibility of spermatozoa to oxidative stress resulting from excessive reactive oxygen species (ROS) production during spermatogenesis (Bisht et al. 2017; Takeshima et al. 2021). In normal physiological situation, ROS including the superoxide anion and hydrogen peroxide (H_2_O_2_) play a beneficial role in sperm capacitation and acrosome reaction (Du Plessis et al. 2015; O’Flaherty 2020). However excessive production of ROS enhances the oxidative stress, inducing pathological conditions in reproductive processes specifically effecting the overall spermatogenic parameters (Agarwal and Bui 2017; Barati et al. 2020). Earlier studies indicated that excessive ROS production decreased the spermatic cell energy metabolism, the rapid and progressive sperm motility eventually leading to sperm cell death (Shiva et al. 2011; Dutta et al. 2019). Reports also revealed that oxidative damage and the imbalance in oxidative defense systems during ageing process might negatively effect and appear to be one of the common features in male sexual dysfunctions (Matzkin et al. 2016; Frungieri et al. 2018).

*Cordyceps* (Cordycipitaceae) is a well-known traditional medicinal mushroom with immense biological activities including immunomodulatory, anticancer, antioxidant, anti-inflammatory and antimicrobial activities (Tuli et al. 2014; Olatunji et al. 2018). Out of various species, *Cordyceps militaris* Linn., is widely used in Asian countries, including China and Korea, to maintain health and boost immunity (Tuli et al. 2014). One of the active constituents of *C. militaris*, cordycepin (3′-deoxyadenosine), a purine nucleoside derivative is a valuable medicament and nutrient with immense health benefits (Tuli et al. 2014; Olatunji et al. 2018; Radhi et al. 2021). Recent reports indicated that cordycepin possessed anti-inflammatory, antitumor, neuroprotective and immunomodulatory effects (Nallathamby et al. 2015; Olatunji et al. 2016; Wang et al. 2019; Xu et al. 2019; Lee et al. 2020; Jin et al. 2021). In relation to reproductive health benefits, cordycepin has been proved to enhance sexual function, treat erectile dysfunction, and acts as a sexual agonist in male reproductive problems (Chen et al. 2017). Previous studies from our laboratory also showed that cordycepin attenuated age-related oxidative stress and sexual dysfunction by improving the sperm kinematics, altering the antioxidative enzyme status, and reproductive hormones in experimental aged rats (Ramesh et al. 2012; Kopalli et al. 2019). However, the beneficial effects of cordycepin related to oxidative stress-induced changes in Leydig cells and the spermatogenic molecular aspects involved have not been reported.

It is well documented that exogenous treatment of H_2_O_2_ has been shown to increase intracellular ROS levels in male reproductive cells (Shi et al. 2018; Pintus and Ros-Santaella 2021). Further, the mouse TM3 Leydig cell lines were well used as a valuable *in vitro* model to study the spermatogenic parameters induced by various cytotoxins including H_2_O_2_ (Ding et al. 2017; Yin et al. 2017; Greifová et al. 2020). Recently, H_2_O_2_ was used as an *in vitro* model to induce ageing (senescence) in TM3 Leydig cells for understanding the molecular mechanisms involving age-related testosterone secretion. Further, H_2_O_2_-induced cellular ageing model in umbilical endothelial vein cells was developed to study the age-related atherosclerotic diseases (Du et al. 2019; Zhang et al. 2020). Ageing is a very complex phenomenon, and oxidative stress with the production of excessive free radicals have been suggested to be involved in the ageing process. In light of such reports, in the present study, we evaluated the effect of cordycepin against H_2_O_2_-induced alterations in oxidative, spermatogenic gene expression and hormonal status in mouse TM3 Leydig cells *in vitro* and further assessed the ageing-mediated antioxidative and apoptosis signalling in aged rats *in vivo*.

## Materials and methods

### Chemicals and reagents

**Figure 1.**
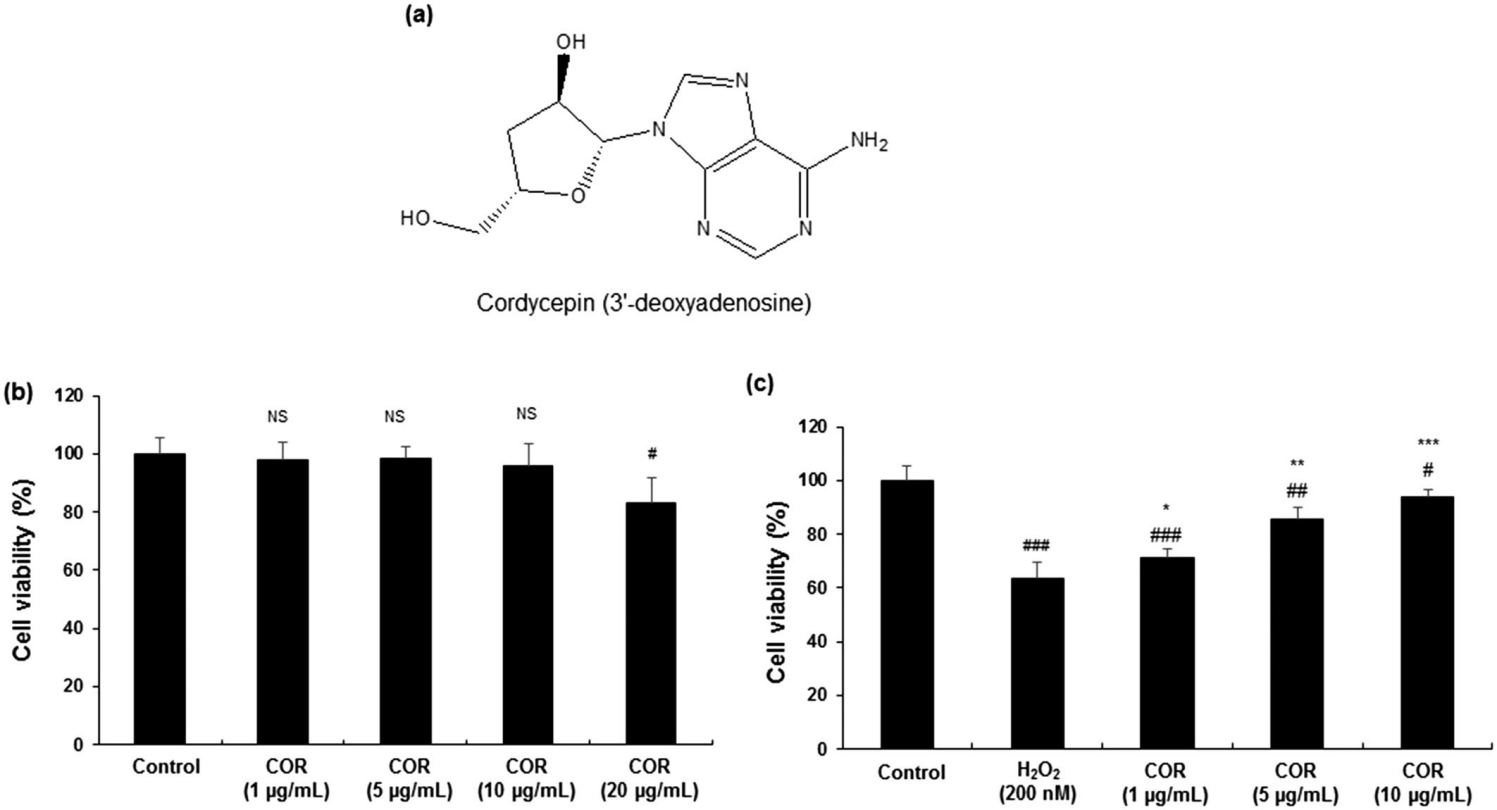
Effect of Cordycepin (COR) on the viability of mouse Leydig TM3 cells. (a): The chemical structure of COR. (b): The cell viability in COR treated TM3 cells. (c): The effect of COR on hydrogen peroxide (H_2_O_2_)-induced TM3 cells. TM3 cells were treated with indicated concentrations of COR in the presence or absence of 200 μM H_2_O_2_ at 37 °C for 24 h. Cell viability was evaluated using the MTT assay and the results are shown as percentage of the control samples. Data are expressed as the mean ± SD (*n* = 6). ^#^*p* < 0.05, ^##^*p* < 0.01 and ^###^*p* < 0.001 compared with control. *p < 0.05, **p < 0.01 and ***p < 0.001, compared to cells exposed to H_2_O_2_.

Cordycepin (COR, 99.5%; Figure 1(A)) was isolated from *C. militaris* as reported previously (Kopalli et al. 2019). Briefly, COR was isolated and purified by butanol partition, silica gel flash chromatograph and recrystallization. Dulbecco’s modified Eagle’s medium (DMEM), foetal bovine serum (FBS), polyvinylidene fluoride (PVDF) membrane, dimethyl sulfoxide (DMSO), H_2_O_2_, and other reagents were purchased from Sigma-Aldrich (St. Louis, MO, USA).

### *In vitro* studies

#### Cell culture and cell viability

Mouse Leydig TM3 cell lines were obtained from the American Type Culture Collection (Manassas, VA, USA). TM3 cells were maintained in DMEM and Ham’s F12 medium (1:1, v/v), supplemented with 5% horse serum (HS, Thermo-Fisher Inc., Rockford, IL, USA) and 2.5% FBS. All media contained 1.2 g/L sodium bicarbonate, 15 mM HEPES, 100 IU/mL penicillin, and 100 mg/mL streptomycin.

For viability tests, MTT [3-(4,5-dimethylthiazol-2-yl)-2,5-diphenyltetrazolium bromide] assay was used. The principle involves MTT, a substrate of mitochondrial enzyme succinate dehydrogenase (yellow initially) is capable of cleaving certain covalent bonds of MTT transforming it into formazan salt (purple salt) which is insoluble in aqueous solution. The reaction can be monitored quantitatively by spectrophotometry to estimate the cell viabilities. Briefly, the cells were seeded at 1.0 × 10^4^ cells/well on 96-well plate containing appropriate media overnight. COR was treated at the concentration of 1, 5, 10, and 20 μg/mL for 24 h. After addition of 10 μL MTT solution (5 mg/mL), the cells were subsequently incubated at 37 °C for 4 h. The formazan crystals formed in the viable cells were dissolved in 100 μL dimethyl sulfoxide (DMSO) after the medium of the wells was removed. The optical density (OD) was read at 570 nM using a microplate reader (Tecan, Männedorf, Switzerland). All experiments were carried out in triplicate and repeated twice independently. To determine the effect of COR on H_2_O_2_-induced cytotoxicity in TM3 cells, the cells were pre-treated with COR at the indicated concentrations (1, 5 and 10 μg/mL) for 24 h prior to exposure to H_2_O_2_ (200 nM) for 30 min and cell viability was measured using the MTT assay as mentioned above.

### Animals

#### Experimental design

Male Sprague-Dawley rats (twelve, 14-month-old 650 ± 20 g and six, 2-month-old 280 ± 20 g) were procured from Hanil Experimental Animal Breeding Co. Ltd. (Yeumsung, Chungbuk, Korea). The animals were maintained at specific pathogen free environment at the Regional Innovation Centre Experimental Animal Facility, Konkuk University, Republic of Korea at a constant temperature (23 ± 2 °C) and relative humidity (55 ± 10%) on a 12 h light/dark cycle. Rats were acclimatized for at least one week prior to the experiments and were provided a standard pellet diet and water *ad libitum*. Rats were randomly divided into three groups (*n* = 6): the young control (YC) and aged control (AC) groups received vehicle (distilled water) only, whereas the COR-treated aged rat group (COR 20) was administrated COR orally after pelletization at daily dose 20 mg/kg body weight, respectively, for six months. The dose selection and administration procedures were performed based on our previous study (Kopalli et al. 2019). After the last schedule of the experiment, the animals were fasted overnight (only water was provided *ad libitum*), and sacrificed with carbon dioxide according to National Institutes of Health (NIH) guidelines. The YC group was 8 months old and AC/COR 20 groups were 20 months old at the time of sacrifice. The age selection of rats for YC group and AC/COR 20 groups was based earlier report (Sengupta 2013). All animal experiments were performed in accordance with the Institutional Animal Care and Use Committee guidelines of Konkuk University (protocol number ku0808), and the study was approved by the Animal Ethics Committee in accordance with the 14th article of the Korean Animal Protection Law. The testes were isolated after removing any adhering adipose tissue. The testes were washed in ice-cold saline, and the adhering fat and connective tissues were cleaned and removed. A 10% homogenate of the testis tissue was prepared in Tris-HCl buffer (0.1 M, pH 7.4), centrifuged (2500 rpm for 10 min at 4 °C) to pellet the cell debris, and the clear supernatant was used for Western blotting and other biochemical enzymatic and non-enzymatic antioxidative assays.

### Measurement of lipid peroxidation (LPO)

To determine the LPO, indicated concentrations of COR (1, 5 and 10 µg/mL) and H_2_O_2_ (200 nM) were added to each well in accordance with the experimental design used for estimating cell viabilities. The cell plates were incubated for 24 h. Lipid peroxidation was determined by the procedure as reported earlier (Ohkawa et al. 1979). One of the major secondary products of lipid peroxidation is malondialdehyde (MDA) which readily reacts with thiobarbituric acid (TBA) to generate a coloured product, thiobarbituric acid reactive substances (TBARS) with maximum absorption at 532 nM. The concentration of TBARS was calculated from a standard calibration curve generated with known amounts of MDA expressed as nM per mg protein.

### Determination of testosterone level

Testosterone levels were determined by adding equal volumes of indicated concentrations of COR (1, 5, and 10 µg/mL) and H_2_O_2_ (200 nM) to each well in accordance with procedure followed in cell viability assay, and the plate was incubated for 24 h. The testosterone levels in the medium were measured using an enzyme immunoassay kits (Assay Designs, MI, USA) following the manufacturer’s instructions.

### Determination of enzymatic and non-enzymatic antioxidant status

To determine the enzymatic antioxidant levels, the activities of superoxide dismutase (SOD), catalase (CAT), glutathione peroxidase (GPx), glutathione reductase (GR) and glutathione-*S*-transferase (GST) in the testis tissue of young, aged and COR (20 mg/kg)-treated aged rats was evaluated. For non-enzymatic antioxidant status, reduced glutathione (GSH), ascorbic acid (vitamin C) and α-tocopherol (vitamin E) levels were measured. The levels of all the antioxidants mentioned were evaluated as described in our previous report (Kopalli et al. 2015).

### Western blot analysis

Equal amounts of protein from each sample were separated with 10% sodium dodecyl sulphate polyacrylamide gel electrophoresis and were transferred to a polyvinylidene fluoride membrane (Millipore, Billerica, MA, USA). Each membrane was incubated for 1 h in Tris-buffered saline containing 0.1% Tween-20 and 5% skimmed milk to block non-specific antibody binding. The membranes were subsequently incubated with specific primary antibodies (1:2,000 dilutions; Santa Cruz Biotech, Santa Cruz, CA, USA) as described in our previous report (Kopalli et al. 2019). Beta-actin was used as an internal control. Each protein was detected using horseradish peroxidase-conjugated secondary antibodies and a chemiluminescence detection system (GE Healthcare Life Sciences, Little Chalfont, UK).

### RNA isolation and real time reverse transcription polymerase chain reaction (RT-PCR)

RNA-Bee reagent (AMS Bio, Abingdon, UK) was used to extract the total RNA according to the manufacturer’s instructions. RNA (1 µg) was reverse transcribed for 50 min at 37 °C in a mixture containing 1 µL oligo (dT), 10 mM dNTP, 0.1 M dithiothreitol, 5 X polymerase chain reaction (PCR) buffer, and 1 µL Moloney Murine Leukaemia Virus Reverse Transcriptase (RT) (Invitrogen Co., Carlsbad, CA, USA). An aliquot (200 ng) of the RT products was amplified in a 25 µL reaction volume using a GoTaq^®^ Green Master Mix (Promega Co., Madison, WI, USA) in the presence of 10 pM oligonucleotide primer. The primers used for the RT products was shown in Table 1. The PCR was performed as described in our previous report (Kopalli et al. 2016). The intensity of the bands was normalized to glyceraldehyde-3-phosphate dehydrogenase (GAPDH) and analysed using the ImageJ software package (version 1.41o; National Institutes of Health, Bethesda, MA, USA).

**Table 1.** Primers used in the study.

Peroxiredoxin (PRx) 3	Forward: 5′- ACT TTA AGG GAA AAT ACT TGG TGC T-3′ Reverse: 5′- TCT CAA AGT ACT CTT TGG AAG CTG T-3′
Glutathione-*S*-transferase (GST) m5	Forward: 5′-TAT GCT CCT GGA GTT TAC TGA TAC C-3′ Reverse: 5′-AGA CGT CAT AAG TGA GAA AAT CCA C-3′
Glutathione peroxidase (GPx) 4	Forward: 5′-GCA AAA CCG ACG TAA ACT ACA CT-3′ Reverse: 5′-CGT TCT TAT CAA TGA GAA ACT TGG T-3′
Inhibin-α	Forward: 5′-AGG AAG GCC TCT TCA CTT ATG TAT T-3′ Reverse: 5′-CTC TTG GAA GGA GAT ATT GAG AGC-3′
Nectin-2	Forward: 5′- CAC TAT CAT CAG CCG ATA CTC CT-3′ Reverse: 5′- GCT GTA CAG ATG AAG GTA GTG TTG A-3′
Bax	Forward: 5′-ATG-GAC-GGG-TCC-GGG-GAG-3′ Reverse: 5′-TGG-AAG-AAG-ATG-GGC-TGA-3′
Bcl-2	Forward: 5′-CAG-CTG-CAC-CTG-ACG-3′ Reverse: 5′-GCT-GGG-TAG-GTG-CAT-3′
p53	Forward: 5′-GCT-CTG-ACT-GTA-CCA-CCA-TCC-3′ Reverse: 5′-CTC-TCG-GAA-CAT-CTC-GAA-GCG-3′
Glyceraldehyde-3-phosphate dehydrogenase (GAPDH)	Forward: 5′-AAC TTT GGC ATT GTG GAA GGG C-3′ Reverse: 5′-ACA CAT TGG GGG TAG GAA CAC G-3′

### Statistical analysis

The data were expressed as the mean ± standard deviation (SD). Statistical evaluation of the data was performed by one-way analysis of variance (ANOVA) followed by Tukey’s *post hoc* test for multiple comparisons using the Graph-Pad prism software package (version 6.0; GraphPad, Inc., La Jolla, CA, USA) for Windows. A value of *p <* 0.05 was considered statistically significant.

## Results

### Effect of COR on cell viability and H_2_O_2_-exposed cytotoxicity in TM3 cells

To evaluate the effect of COR alone on the TM3 cell viability, various concentration of COR (1, 5, 10, and 20 μg/mL) were incubated with TM3 cells for 24 h. Treatment with COR alone up to 10 μg/mL did not affect or influenced the over cell viability in TM3 cells observed by MTT assay. COR treatment at 20 μg/mL concentration showed cytotoxic effects and therefore the non-toxic concentration of 1, 5 and 10 μg/mL were used for further studies (Figure 1(b)).

The effect of COR on H_2_O_2_-induced cytotoxicity in TM3 cells was shown in Figure 1(c). TM3 cells treated with H_2_O_2_ (200 μM) exhibited significant (*p <* 0.001) cytotoxic effects and decreased the overall percentage of cell viability (63.27 ± 6.12%). Treatment with indicated concentrations of COR significantly (*p <* 0.05, *p <* 0.01and *p <* 0.001 at 1, 5, and 10 μg/mL, respectively) ameliorated the cytotoxic effect of H_2_O_2_ in TM3 cells with maximum effects observed at 10 μg/mL concentration ((71.25 ± 3.24%, 85.67 ± 4.12% and 93.97 ± 2.89% at 1, 5, and 10 μg/mL).

### Effect of COR on the expression level of key antioxidant enzymes in H_2_O_2_-exposed TM3 cells

**Figure 2.**
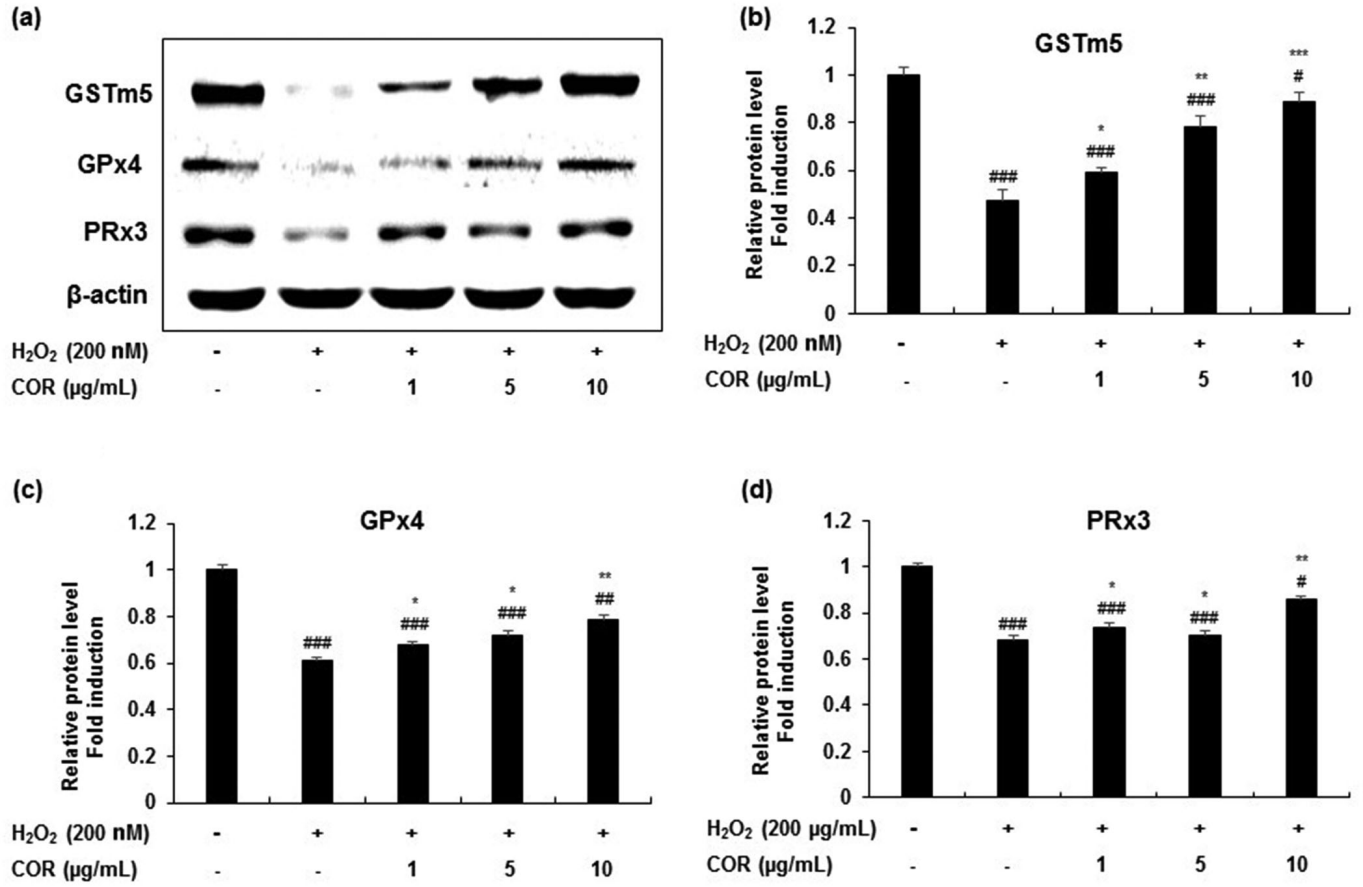
The effect of cordycepin (COR) on the protein expression of antioxidant enzyme in hydrogen peroxide (H_2_O_2_)-induced TM3 cells. (a) Protein expression of GSTm5, GPx4, and PRx3 analysed by western blotting. (b-d) Relative expression levels (fold) of GSTm5, GPx4, and PRx3 in three independent experiments, respectively. β-Actin was used as an internal control. The data are expressed as the mean ± SD (*n* = 6). ^#^*p* < 0.05, ^##^*p* < 0.01 and ^###^*p* < 0.001 compared with control. *p < 0.05, **p < 0.01 and ***p < 0.001, compared to cells exposed to H_2_O_2._ GSTm5: glutathione-*S*-transferase m5, GPx4: glutathione peroxidase 4 and PRx3: peroxiredoxin 3.

To understand the effect of COR on H_2_O_2_-induced changes in oxidative enzyme status, the expression of key spermatogenesis-related antioxidant enzymes such as GSTm5, GPx4 and PRx3 were analysed. COR at indicated concentrations (1, 5, and 10 μg/mL), markedly ameliorated the H_2_O_2_-induced decrease in the expression level of antioxidant enzymes such as GSTm5, GPx4 and PRx3 (Figure 2(a)). Quantification data revealed a significant decrease in the protein expression of GSTm5, GPx4 and PRx3 in H_2_O_2_-treated TM3 cells (*p <* 0.001). However, COR treatment significantly attenuated the decreased protein expression of GSTm5 (*p <* 0.05, *p <* 0.01, and *p <* 0.001 at 1, 5, and 10 μg/mL), GPx4 (*p <* 0.05 at 1 and 5 μg/mL; *p <* 0.01 at 10 μg/mL) and PRx3 (*p <* 0.05 at 1 and 5 μg/mL; *p <* 0.01 at 10 μg/mL) enzymes showing a maximum effect at 10 μg/mL concentration (Figure 2(b–c)).

**Figure 3.**
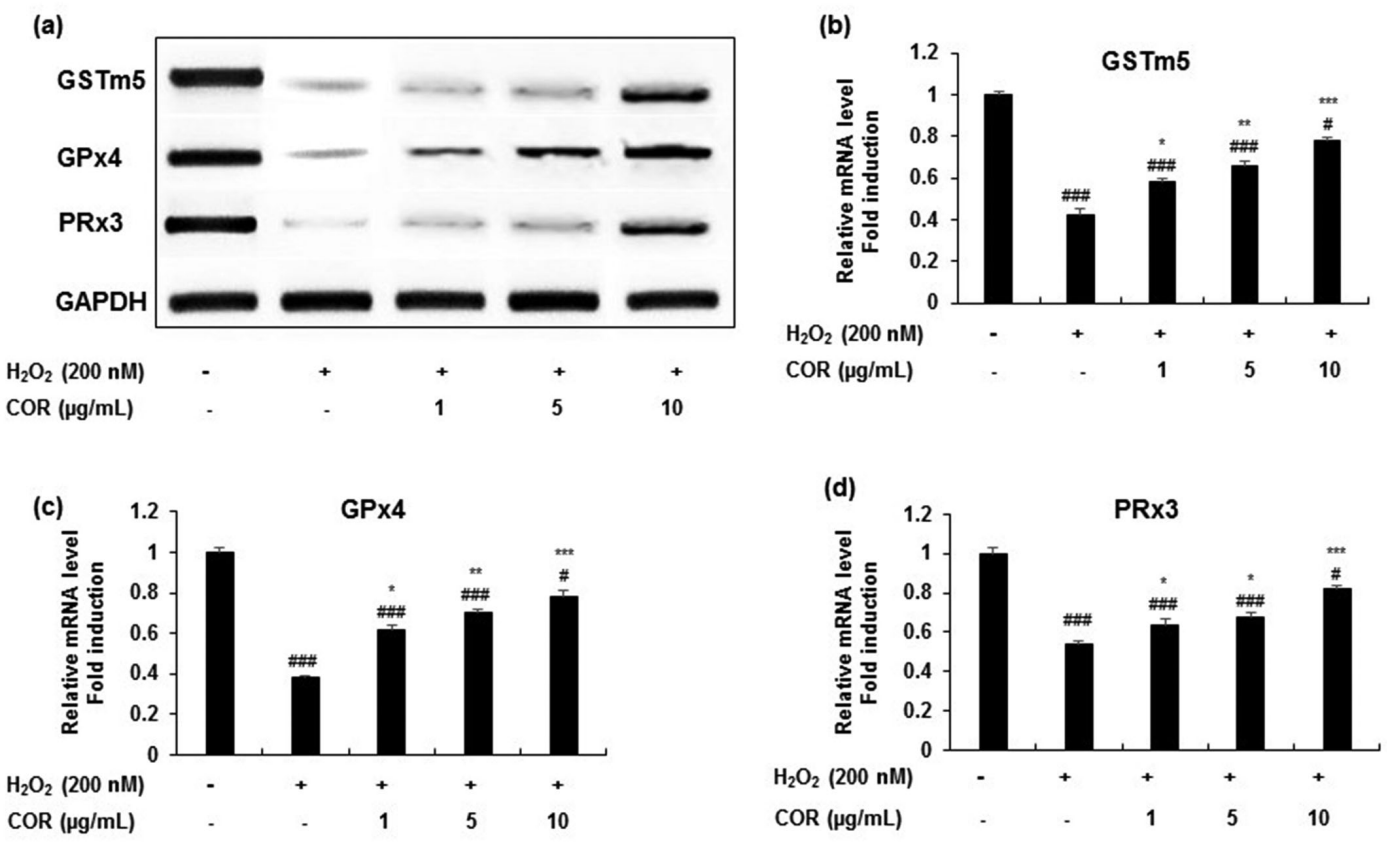
The effect of COR on the mRNA expression of antioxidant enzyme in hydrogen peroxide (H_2_O_2_)-induced TM3 cells. (a): The mRNA expression of GSTm5, GPx4, and PRx3 analysed by RT-PCR. (b-d): Relative expression levels (fold) of GSTm5, GPx4, and PRx3 in three independent experiments, respectively. GAPDH was used as an internal control. The data are expressed as the mean ± SD (*n* = 6). ^#^*p* < 0.05 and ^###^*p* < 0.001 compared with control. *p < 0.05, **p < 0.01 and ***p < 0.001, compared to cells exposed to H_2_O_2._ COR: cordycepin, GSTm5: glutathione-*S*-transferase m5, GPx4: glutathione peroxidase 4 and PRx3: peroxiredoxin 3, GAPDH: Glyceraldehyde 3-phosphate dehydrogenase, RT-PCR: reverse transcriptase-polymerase chain reaction.

Similar results were obtained in the mRNA expression (Figure 3(a)) with significant attenuation in the decreased levels of GSTm5 (*p <* 0.05, *p <* 0.01, and *p <* 0.001 at 1, 5, and 10 μg/mL), GPx4 (*p <* 0.05, *p <* 0.01 and *p <* 0.001 at 1, 5, and 10 μg/mL) and PRx3 (*p <* 0.05 at 1 and 5 μg/mL; *p <* 0.001 at 10 μg/mL) in TM3 cells treated with H_2_O_2_ (Figure 3(b–d)). These results suggest that COR exhibited potential antioxidant ability by LPO inhibition and modulated the expression of key antioxidant-related genes such as GSTm5, GPx4 and PRx3 in H_2_O_2_-induced oxidative damage in TM3 cells.

### Effect of COR on LPO production in H_2_O_2_-exposed TM3 cells

**Figure 4.**
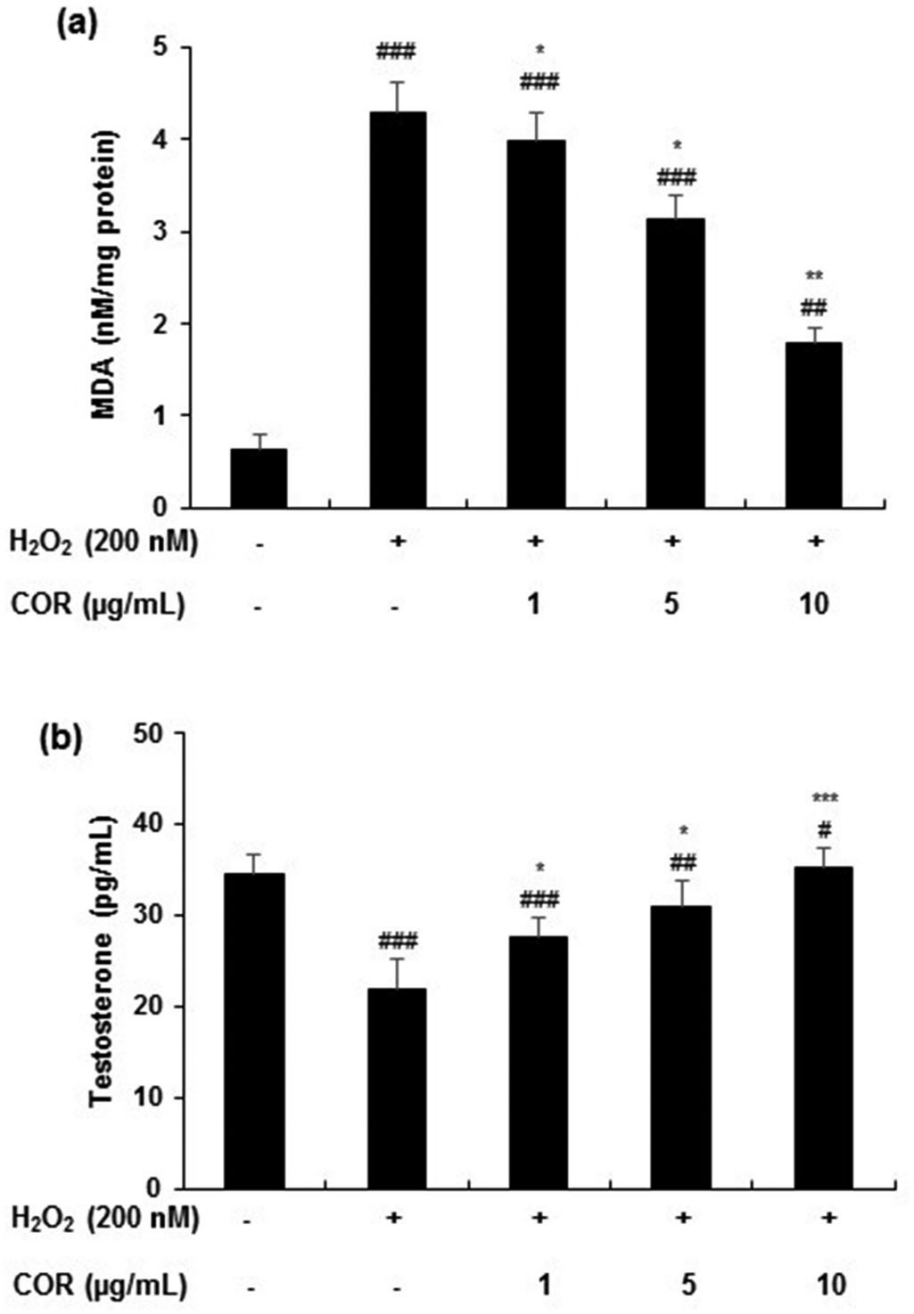
Effect of cordycepin (COR) on lipid peroxidation and testosterone levels in hydrogen peroxide (H_2_O_2_)-induced TM3 cells. (a) Effect of COR on H_2_O_2_-induced lipid peroxidation in TM3 cells. TM3 cells treated with COR were incubated in the presence or absence of 200 μM H_2_O_2_ at 37 °C for 24 h. Total cell lysate from cultured cells was analysed for malondialdehyde (MDA) formation. (b): Effect of COR on H_2_O_2_-induced testosterone production in TM3 cells. Data are expressed as the mean ± SD (*n* = 6). ^#^*p* < 0.05, ^##^*p* < 0.01 and ^###^*p* < 0.001 compared with control. *p < 0.05, **p < 0.01 and ***p < 0.001, compared to cells exposed to H_2_O_2_.

The LPO inhibition effect of COR on H_2_O_2_-induced TM3 cells was analysed through the formation of MDA (Figure 4(a)). H_2_O_2_ treated cells showed a significant increase (*p <* 0.001) in MDA levels (4.28 ± 0.35 nM/mg protein) when compared with control levels (0.64 ± 0.15 nM/mg protein). However, COR treatment (1, 5, and 10 µg/mL) significantly reduced the MDA production to 3.98 ± 0.31 (*p <* 0.5), 3.14 ± 0.26 (*p <* 0.5) and 1.78 ± 0.18 (*p <* 0.01) nM/mg protein, respectively.

### Effect of COR on the testosterone production in H_2_O_2_-exposed TM3 cells

The levels of testosterone production in H_2_O_2_-induced TM3 cells were significantly reduced compared with control cells (from 34.45 ± 2.24 pg/mL to 21.89 ± 3.21 pg/mL, *p <* 0.001). However, COR treatment to H_2_O_2_-induced TM3 cells increased the testosterone production (27.68 ± 1.98, 30.89 ± 2.86 and 35.24 ± 2.18 at 1, 5, and 10 µg/mL) significantly (*p <* 0.05, *p <* 0.01 and *p <* 0.001, respectively) (Figure 4(b)).

### Effect of COR on the expression of spermatogenesis-related genes in H_2_O_2_-exposed TM3 cells

**Figure 5.**
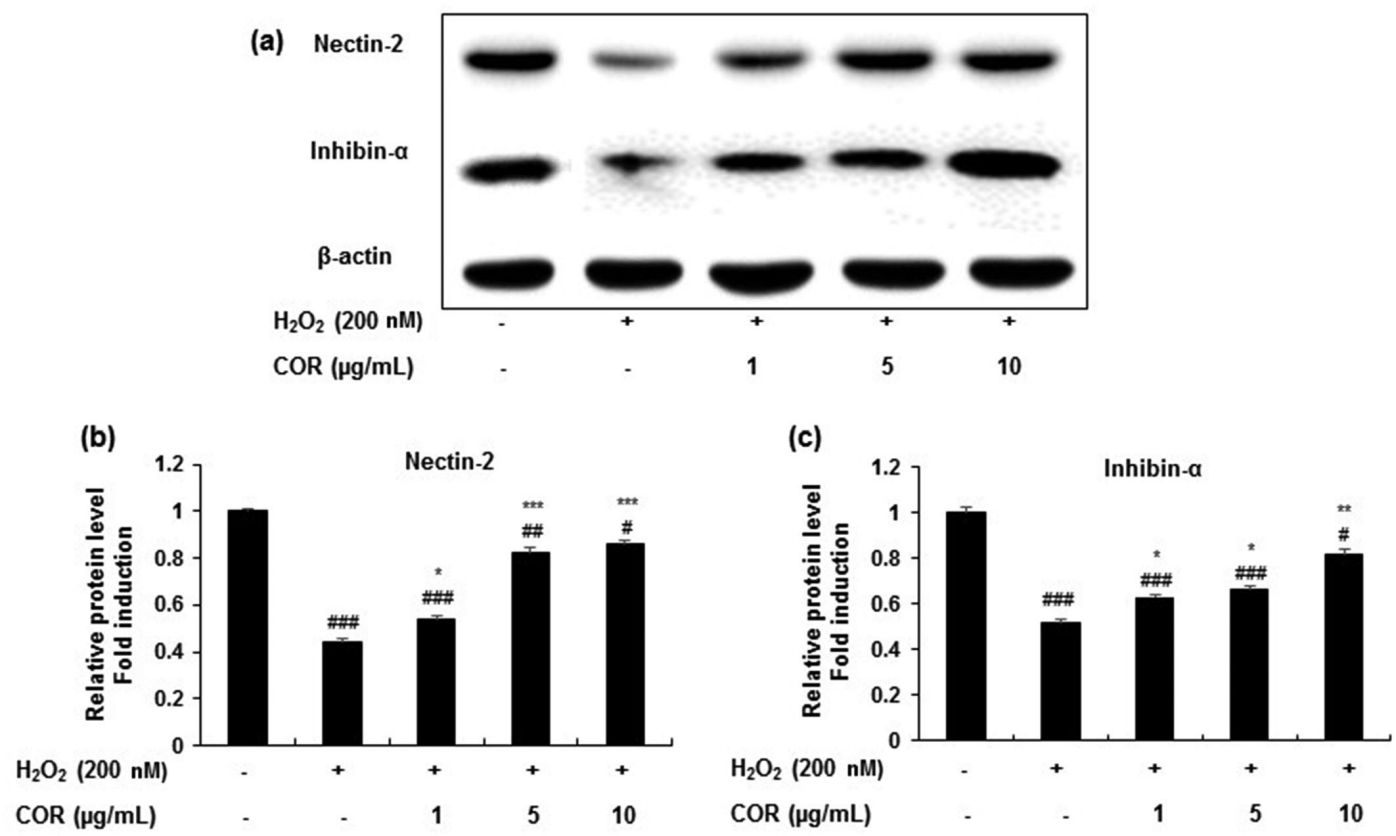
The effect of cordycepin (COR) on the protein expression of spermatogenesis-related molecules and in hydrogen peroxide (H_2_O_2_)-induced TM3 cells. (a): Protein expression of nectin-2, and inhibin-α analysed by western blotting. (b, c): Relative expression levels (fold) of nectin-2, and inhibin-α in three independent experiments, respectively. β-actin was used as an internal control. The data are expressed as the mean ± SD (*n* = 6). ^#^*p* < 0.05, ^##^*p* < 0.01 and ^###^*p* < 0.001 compared with control. *p < 0.05, **p < 0.01 and ***p < 0.001, compared to cells exposed to H_2_O_2_.

**Figure 6.**
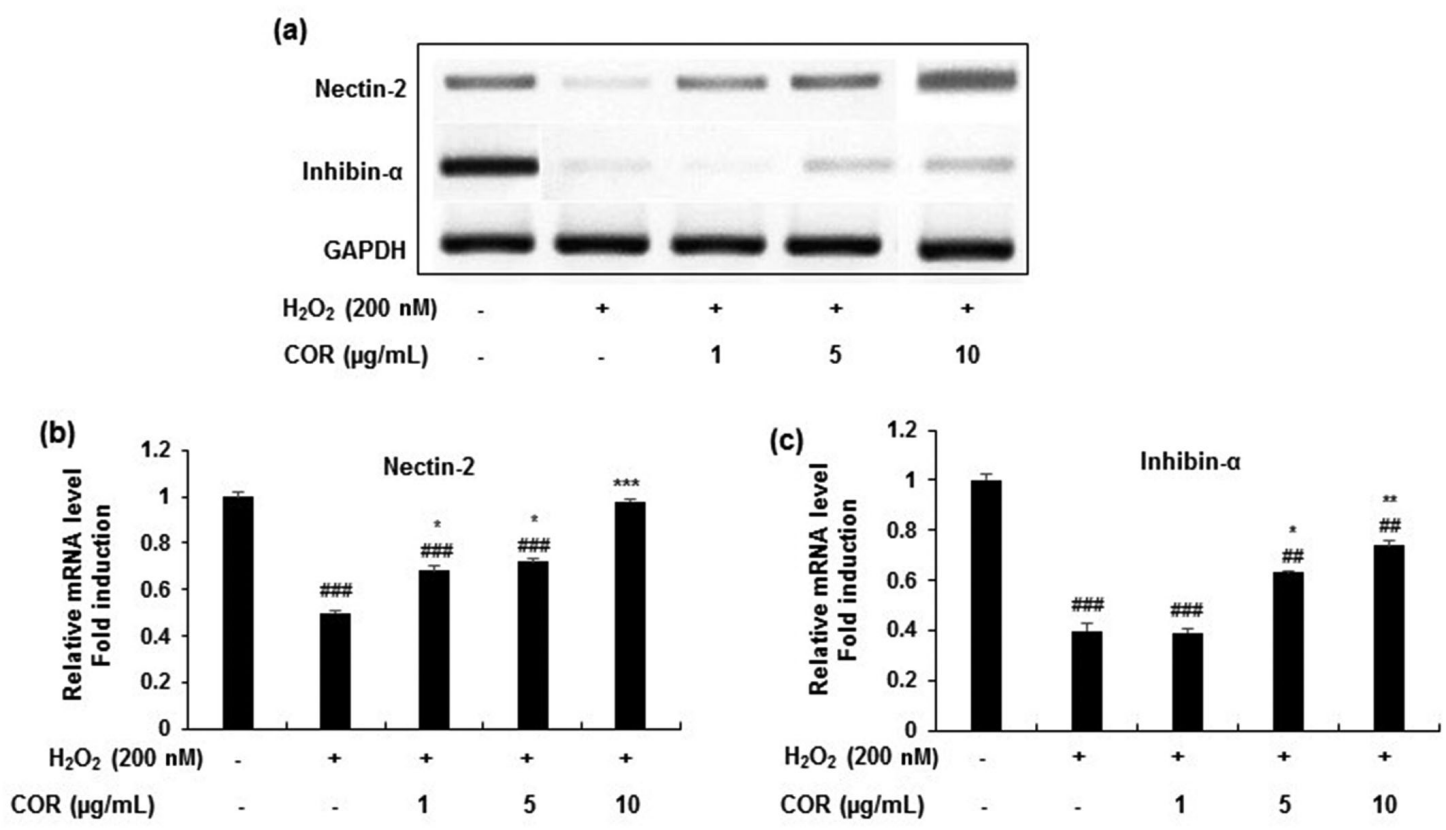
The effect of cordycepin (COR) on the mRNA expression of spermatogenesis-related molecules and in hydrogen peroxide (H_2_O_2_)-induced TM3 cells. (a): The mRNA expression of nectin-2, and inhibin-α analysed by RT-PCR. (b-d): Relative expression levels (fold) of nectin-2, and inhibin-α in three independent experiments, respectively. GAPDH was used as an internal control. The data are expressed as the mean ± SD (*n* = 6). ^##^*p* < 0.01 and ^###^*p* < 0.001 compared with control. *p < 0.05, **p < 0.01 and ***p < 0.001, compared to cells exposed to H_2_O_2_. GAPDH: Glyceraldehyde 3-phosphate dehydrogenase, RT-PCR: reverse transcriptase-polymerase chain reaction.

To evaluate the effect of COR on spermatogenesis-related genes the protein and mRNA expression of nectin-2 and inhibin-α were analysed in H_2_O_2_-treated TM3 cells (Figures 5 and 6). Results showed that the protein expression levels of nectin-2 and inhibin-α in H_2_O_2_-exposed TM3 cells were markedly reduced and treatment with COR at indicated concentrations (1, 5, and 10 μg/mL) restored the decreased protein expression (Figure 5(a)). Quantification data revealed that treatment with H_2_O_2_ to TM3 cells significantly (*p <* 0.001) decreased the protein expression of nectin-2 and inhibin-α (Figure 5(b–c)). However, COR at indicated concentrations (1, 5, and 10 µg/mL) significantly ameliorated the decreased protein expression of both of nectin-2 (*p <* 0.05, *p <* 0.001 and *p <* 0.001 at 1, 5, and 10 μg/mL, respectively) and inhibin-α (*p <* 0.05, *p <* 0.05 and *p <* 0.01 at 1, 5, and 10 μg/mL, respectively). A similar pattern was observed in the mRNA expression in both nectin-2 and inhibin-α in TM3 cells treated with H_2_O_2_ exhibiting a maximum effect at 10 µg/mL concentration (Figure 6(a–c)). These results suggest that COR ameliorated the expression levels of key molecules of spermatogenesis-related genes in H_2_O_2_-induced TM3 cells.

### Effect of COR on enzymatic and non-enzymatic antioxidant levels in young and aged rat testis

**Figure 7.**
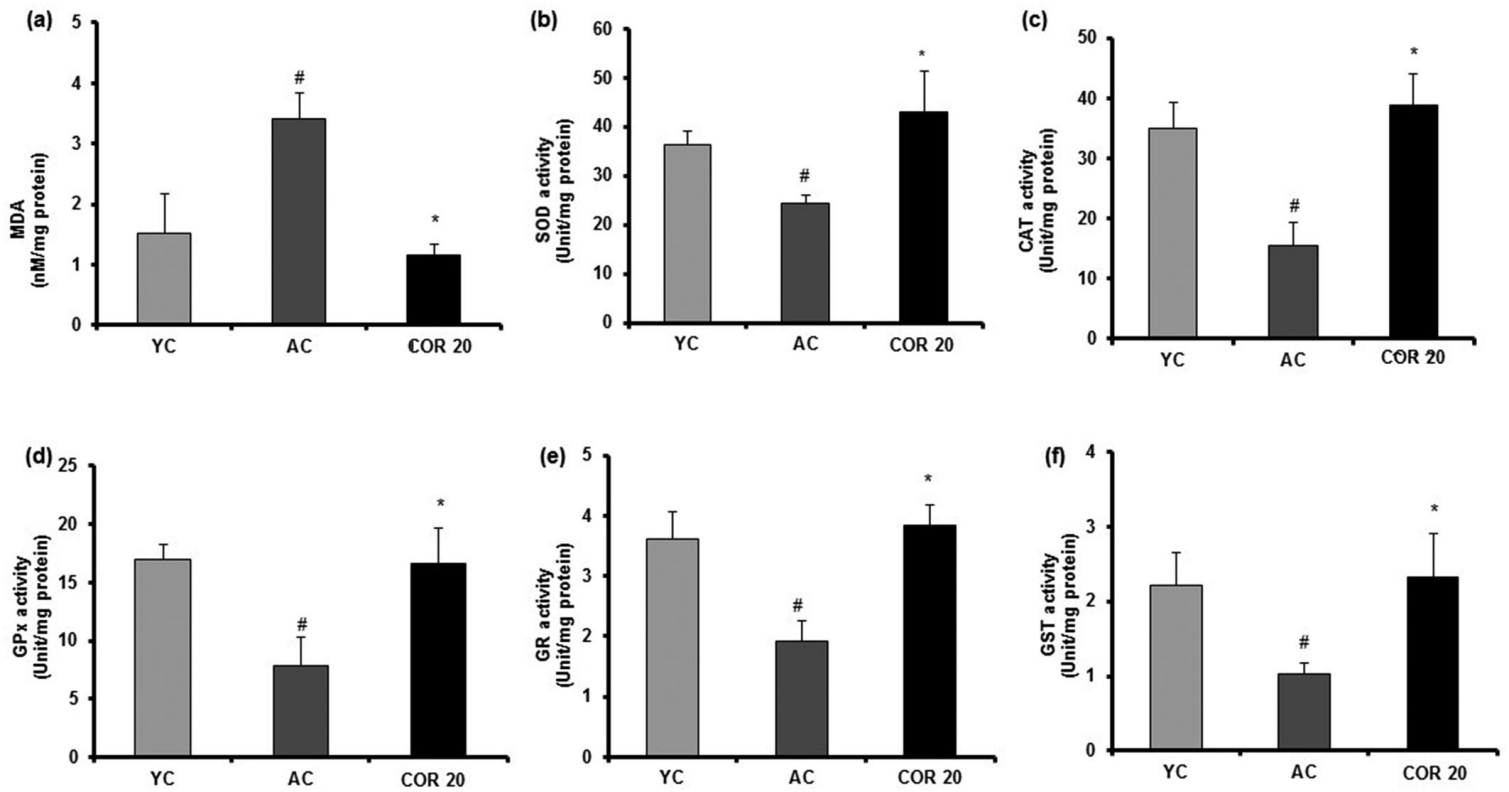
Effect of COR on testis lipid peroxidation (LPO) and enzymatic levels in aged rats. MDA level (a), SOD level (b), CAT level (c), GPx level (d), GR level (e) and GST level (f). The results are expressed as mean ± SD (*n* = 6), where ^#^*p* < 0.05 compared with YC group, *p < 0.05 compared with AC group. YC: young rats; AC: aged rats; COR 20: cordycepin (COR) 20 mg/kg treated aged rats; MDA: malondialdehyde, SOD: superoxide dismutase; CAT: catalase; GPx: glutathione peroxidase; GR: glutathione reductase and GST: glutathione-*S*-transferase.

**Figure 8.**
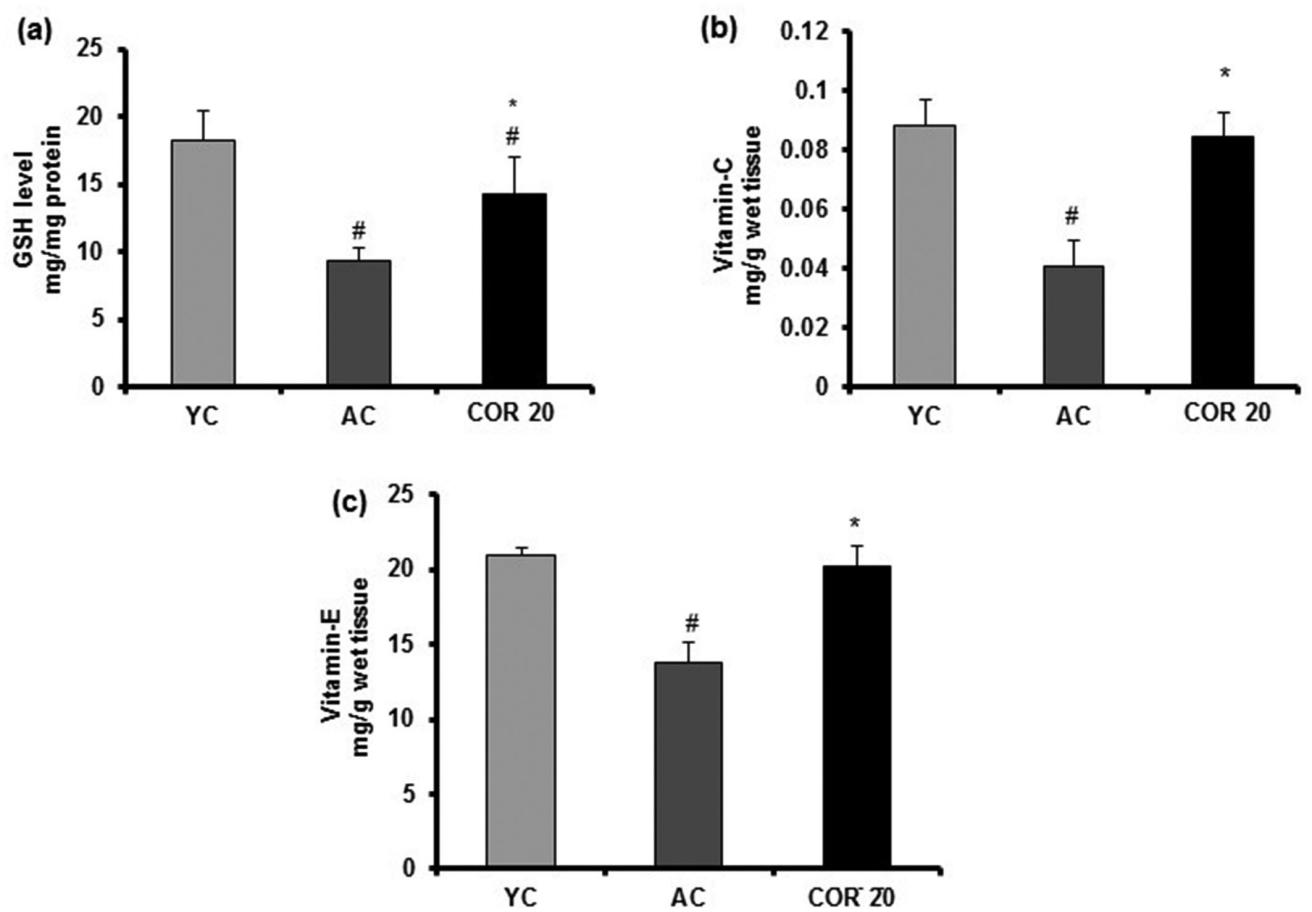
Effect of COR on testis non-enzymatic antioxidant in aged rats. GSH level (a), vitamin C level (b) and vitamin E level (c). The results are expressed as mean ± SD (*n* = 6), where ^#^*p* < 0.05 compared with YC group and *p < 0.05 compared with AC group. YC: young rats; AC: aged rats; COR 20: cordycepin (COR) 20 mg/kg treated aged rats; GSH: reduced glutathione; vitamin C: ascorbic acid and vitamin E: α-tocopherol.

Various testicular oxidation parameters in YC, AC and COR 20 groups were measured in the testis tissues of rats (Figures 7 and 8). Results indicated a significant (*p* < 0.05) 2-fold increase in the lipid peroxidation marker (MDA) in aged rats when compared with YC group indicating that there is an increased lipid peroxidation by ageing (Figure 7(a)). Further, the enzymatic (Figure 7(b–f)); SOD, CAT, GPx, GR and GST) and non-enzymatic (Figure 8(a–c); GSH, vitamin C and vitamin E) antioxidant levels were reduced significantly (*p* < 0.05) in aged rats when compared with YC group. However, treatment with COR (20 mg/kg) to aged rats restored these changes when compared with untreated aged rats significantly (*p* < 0.05). These results indicate that COR exhibited a potential role in ameliorating the altered antioxidative enzyme levels observed in AC groups.

### Effect of COR on apoptotic expression in young and aged rats

**Figure 9.**
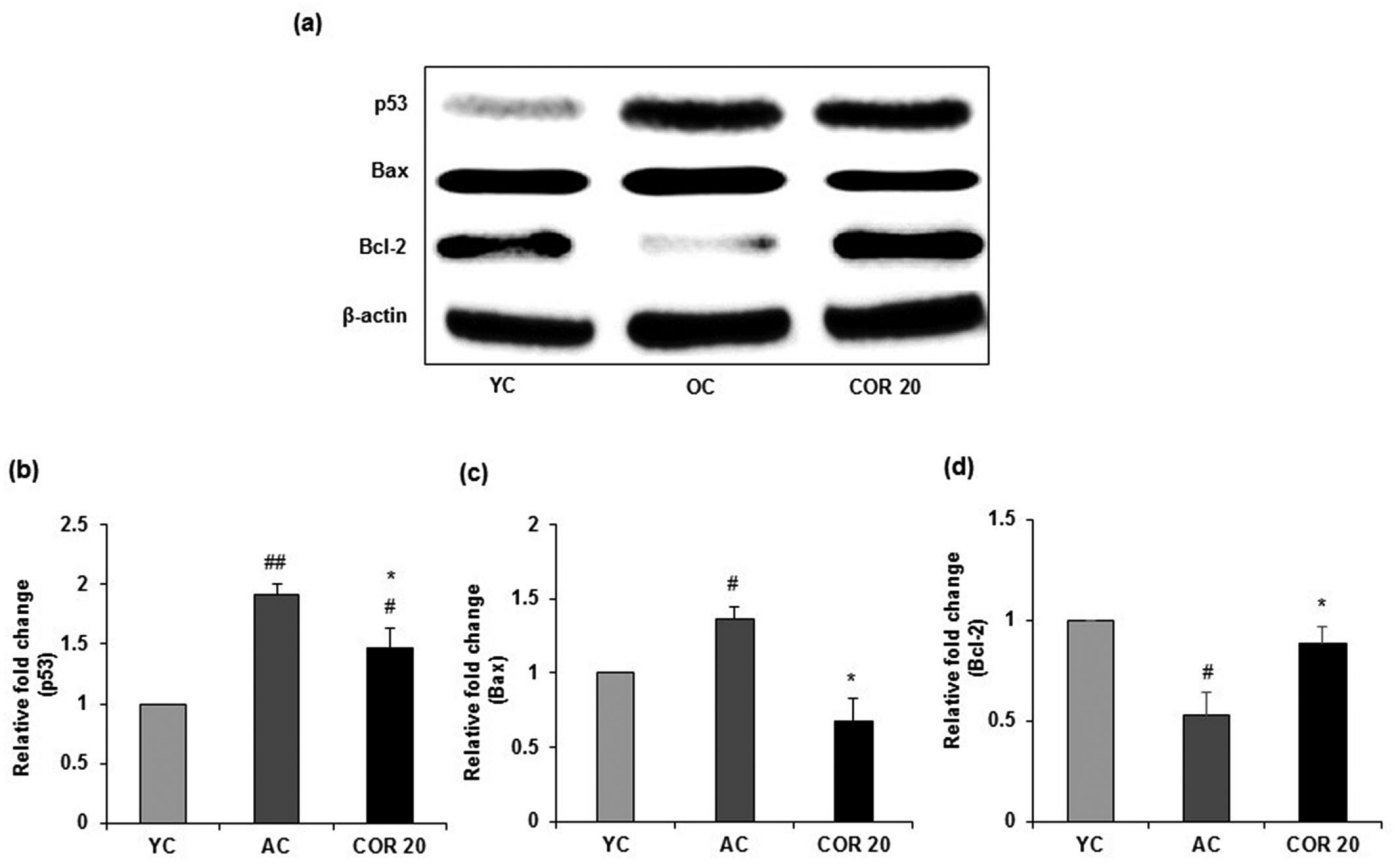
Effect of COR on testicular pro- and anti-apoptotic related protein expression in aged rats. (a) Western blotting analysis of p53, Bax and Bcl-2. Relative expression levels (fold) of p53 (b), Bax (c) and Bcl-2 (d) in three independent experiments, respectively. β-actin was used as an internal control. Data are expressed as the mean ± SD (*n* = 6). ^#^*p* < 0.05 and ^##^*p* < 0.01 compared with YC group and *p < 0.05, compared to AC group. YC: young rats; AC: aged rats; COR 20: cordycepin (COR) 20 mg/kg treated aged rats; Bcl-2: B-cell lymphoma-2; Bax: Bcl-2-associated X.

**Figure 10.**
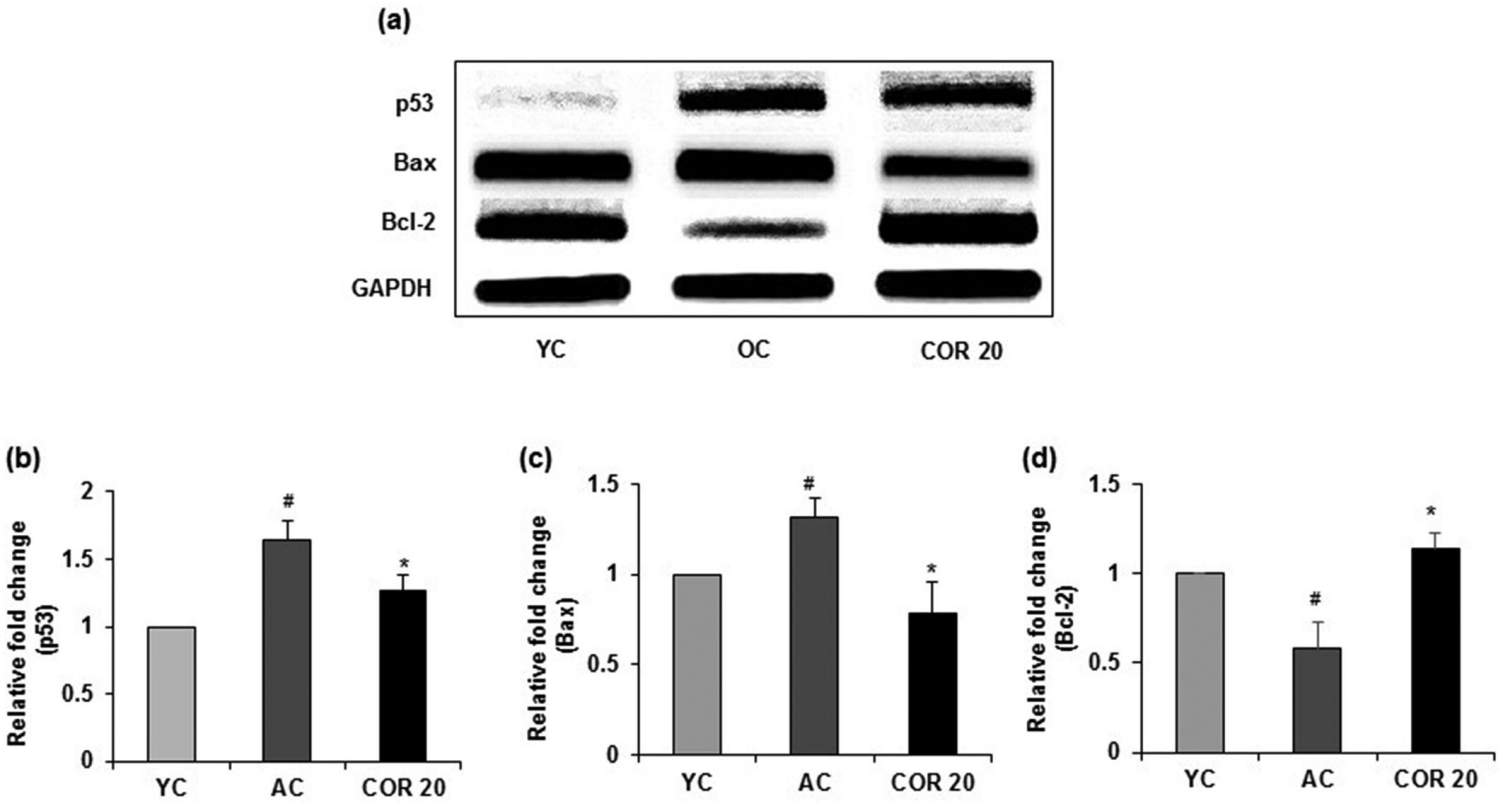
Effect of COR on testicular pro- and anti-apoptotic related mRNA expression in aged rats. RT-PCR analysis of p53, Bax and Bcl-2 mRNA expression (a). Relative expression levels (fold) of p53 (b), Bax (c) and Bcl-2 (d) in three independent experiments, respectively. GAPDH was used as an internal control. Data are expressed as the mean ± SD (*n* = 6). ^#^*p* < 0.05 compared with YC group and *p < 0.05, compared to AC group. YC: young rats; AC: aged rats; COR 20: cordycepin (COR) 20 mg/kg treated aged rats; Bcl-2: B-cell lymphoma-2; Bax: Bcl-2-associated X; RT-PCR: reverse transcriptase-polymerase chain reaction.

To evaluate the effects of COR treatment on ageing-mediated apoptotic signalling, we investigated whether COR alters the activation of pro-apoptotic and anti-apoptotic proteins in aged rats (Figure 9(a)). Data revealed that p53 protein expression was significantly (*p* < 0.01) elevated in aged rats when compared with YC group. However, COR treatment to aged rats significantly (*p* < 0.05) reduced the expression when compared to untreated OC group (Figure 9(b)). Further, the level of Bax was markedly increased (*p* < 0.05) in aged rats when compared with YC group and COR treated OC group suppressed the enhanced expression of Bax (*p* < 0.05; Figure 9(c)). In contrast, the anti-apoptotic protein Bcl-2 was downregulated in aged rat groups compared with YC group but was significantly (*p* < 0.05) upregulated in COR treated aged rats at 20 mg/kg dose (Figure 9(d)). A similar pattern was observed in the mRNA expression in p53, Bax and Bcl-2 in aged rats when comparted with YC group (*p* < 0.05) and these changes in expression was ameliorated with COR treatment significantly (*p* < 0.05; Figure 10(a–d)). These results suggest that COR regulated the ageing-mediated apoptosis related expression levels.

## Discussion

Oxidative damage by excessive generation of ROS has been a primary factor and strongly implicated in the pathology of male infertility (Agarwal and Bui 2017; Barati et al. 2020). Decreased antioxidant capability and increased ROS production exhibit a negative impact on spermatogenic functional parameters (Dutta et al. 2019). Mounting evidence suggests H_2_O_2_ as one of the major ROS generating chemical attacking several components of cells such as proteins, lipids and DNA causing potential oxidative insults (Shi et al. 2018; Pintus and Ros-Santaella 2021). H_2_O_2_ produced excessive reactive hydroxyl radicals are cytotoxic causing sperm kinematic alternations such as declined motility, progression and reduced spermatozoa energy metabolism (Shi et al. 2018).

Mouse Leydig TM-3 cells derived from primary testicular cell cultures and commercially available rodent Leydig cell lines are well documented cell models for male reproductive mechanistic investigations and as screening tools to study testicular steroidogenesis (Kim et al. 2015; Lee et al. 2016; Odermatt et al. 2016). Exposure to H_2_O_2_ at physiological concentrations in Leydig cells exhibited a direct cytotoxic effect and reduced the sperm functions in experimental models (Guesmi et al. 2016; Greifová et al. 2020). Several studies also indicated that H_2_O_2_-induced cytotoxicity in TM3 Leydig cells as a promising *in vitro* oxidative stress and cellular ageing model to evaluate the beneficial effects of various synthetic and natural antioxidant agents in male reproductive dysfunctions (Kim et al. 2015; Wang et al. 2015; Lee et al. 2016; Ding et al. 2017; Sun et al. 2017; Byun et al. 2019; Zhang et al. 2020). Based on the above studies, we examined the protective effects of COR against H_2_O_2_-induced oxidative damage in mouse Leydig TM3 cells. Our present data showed that COR treatment alone up to 10 µg/mL did not affect the overall cell viability in TM3 cells. Further, decreased TM3 cell viability was observed with exposure to H_2_O_2_ and pre-treatment with COR in the presence of H_2_O_2_ significantly restored the decreased cell viability in TM3 cells.

Earlier reports indicated that H_2_O_2_-induced oxidative damage in sperm cells negatively effects the functional and structural properties at various enzymatic and cell signalling receptors (Shi et al. 2018; Greifová et al. 2020). H_2_O_2_ exposure increased the LPO reactions causing membrane damage, loss of ATP ultimately leading to decreased sperm motility and increased morphological defects in sperm cells (Kistanova et al. 2009). In agreement, our results revealed that H_2_O_2_ exposure increased the LPO products by increasing the concentrations of MDA in TM3 cells and COR treatment significantly reduced the formation of TBARS as analysed by MDA levels. Further, studies also revealed that H_2_O_2_ induce oxidative damage and negatively effects steroidogenesis in mouse Leydig cells by altering the antioxidant defense-related enzymes (Wang et al. 2015; Yu and Huang 2015). Therefore, we evaluated the effect of COR on key oxidation-regulating enzymes such as GST-m5, Gpx-4 and PRx3 in TM3 cells exposed to H_2_O_2_. It is well documented that GST-m5, Gpx-4 and PRx3 constitute an important antioxidant protein family with significant roles in various biological processes in several species including humans (Rao and Shaha 2000; Forman and Zhang 2021). In context to male reproductive mechanisms GST-m5, GPx-4 and PRx3 harbour sequence variants in humans, which in turn contribute to the incidence of male infertility especially under environmental ROS oxidative stress (Yu and Huang 2015; Adewoyin et al. 2017). Earlier studies revealed that H_2_O_2_ exposure to spermatozoa significantly reduced the viability and altered the antioxidant enzyme status of GST, PRx and GPx (Noblanc et al. 2011; O’Flaherty and Rico de Souza 2011; Kopalli et al. 2016; Vorobets et al. 2018; Wagner et al. 2018). Therefore, the analysis of antioxidant genes may help understand the roles of antioxidant signalling network in ROS-related male infertility. In the present study, TM3 cells exposed to H_2_O_2_ showed a significant reduction at both protein and mRNA expression of antioxidant-related enzymes, GST-m5, GPx-4 and PRx3. Treatment with COR ameliorated the decreased expression of these enzymes significantly in a dose-dependent fashion. These data indicate that COR might regulate the antioxidant enzyme status against the oxidative insult caused by H_2_O_2_ in TM3 cells.

It is well documented that the major transcriptional factors involved in male reproductive function are the spermatogenesis related proteins namely, nectin-2 and inhibin-α (Mueller et al. 2003; Lee et al. 2019). Nectin-2-deficient male mice exhibited reduced migration of sperm to the oviduct, spermatozoa-zona binding, and sperm-oocyte fusion leading to male infertility (Mueller et al. 2003; Cai et al. 2011). Further, mice lacking nectin-2 gene showed malfunction in the nuclear and cytoskeletal morphology in spermatozoa (Bouchard et al. 2000). In parallel, inhibin a glycoprotein hormone of gonadal origin is responsible for the negative feedback mechanisms that suppress follicle stimulating hormone (FSH) production from the pituitary gland (Kumanov et al. 2005). Several studies revealed that inhibin is a valuable index and a better marker of spermatogenesis in the evaluation of male factor infertility and is one of the major sources in rapid identification of spermatogenic disorders in populations exposed to testicular toxicants (Meachem et al. 2001). Measurement of inhibin is very useful in experimental studies and imparts key information of testicular function in several pathophysiological conditions (Meachem et al. 2001; Cai et al. 2011). In view of the published reports, in our study, H_2_O_2_ treatment decreased the expression of nectin-2 and inhibin-α in TM3 cells at both protein and mRNA levels. These findings indicated that H_2_O_2_-induced oxidative stress might affect the functional and signal transduction pathway involved in spermatogenesis. COR significantly reversed the oxidative stress-induced mRNA and protein expression changes, suggesting that COR might regulate certain key transcription factors and restore signal transduction.

It is well documented that the male reproductive hormone including the FSH, luteinizing hormone and testosterone are well balanced in controlling the male sexual response under normal physiological conditions (Appasamy et al. 2007; Araujo and Wittert 2011; Kavoussi and Costabile 2012). In particular, testosterone produced in the Leydig cells under the influence of luteinizing hormone, is indispensable for sperm production (Oh 2014; Zirkin and Papadopoulos 2018; Ajayi and Akhigbe 2020). Healthy spermatogenic process and germ cell survival are maintained by optimum levels of intratesticular testosterone (Zirkin and Papadopoulos 2018). Imbalance in testosterone production or complete inhibition may lead to improper or complete failure of spermatogenesis (McLachlan et al. 2002; Liu et al. 2016). Several studies from *in vitro*, laboratory, and animal experiments indicated that oxidative stress-induced cell damage has a direct effect on the overall testosterone production (Glade and Smith 2015; Liu et al. 2016). Controlling oxidative stress releases Leydig cells from oxidative inhibition of testosterone synthesis and can improve testosterone status. H_2_O_2_ is known to induce DNA damage and negatively affect the function and secretion of sex hormones including testosterone in mouse testicular cells (Kim et al. 2015; Lee et al. 2016). Accordingly, H_2_O_2_ significantly decreased the testosterone levels in TM3 cells and treatment with COR dose-dependently restored the testosterone levels indicating that COR might play a crucial role in regulating the primary sex hormone functions in developing the male reproductive germ cells.

Earlier reports revealed that *C. militaris* exhibited protective effects against H_2_O_2_-induced oxidative stress and down regulated the inflammation-related proteins in C6 glial cells based on its ROS inhibitory actions indicating the neuroprotective benefits of *C. militaris* against oxidative stress (He et al. 2019). Studies from our laboratory reported that COR from *C. militaris* attenuated age-related male sexual dysfunctions in experimental rats based on its antioxidant effects (Ramesh et al. 2012; Kopalli et al. 2019). In agreement with previous reports, the present *in vitro* results support the notion that COR indeed exhibited potent amelioration in oxidative stress-related male reproductive dysfunctions.

It is well documented that free radicals and oxidative stress have been involved in the ageing process and oxidative stress might play a pivotal role in altering the expression of enzymatic and non-enzymatic antioxidants (Matzkin et al. 2016; Frungieri et al. 2018). Earlier report from our laboratory and others revealed that COR possessed antioxidant effects by scavenging the ROS (Ramesh et al. 2012) in various organ tissues including liver, heart, lung and kidney and attenuated the oxidative related genes in aged rats (Kopalli et al. 2019). To further understand the modulatory effect of COR on increased oxidation in aged rats when compared with young groups, we evaluated the enzymatic and non-enzymatic antioxidative status in COR (20 mg/kg) treated aged rat testes. During various metabolic reactions in the body, free radicals will be produced in abundance and this condition might induce cellular damage by lipid peroxidation during ageing (Rajeswary et al. 2007). Ageing-mediated oxidative stress might even negatively affect the testicular tissue by altering the cellular membrane structures and antioxidative enzyme inactivation ultimately leading to male infertility (Rajesh Kumar and Muralidhara 2002). Therefore, free radical release and its metabolic balance during ageing is a check point to eliminate the adverse effects of ROS in testicular cells and tissue damage. In the present study, the increased levels of LPO observed in aged rats when compared with young groups was significantly ameliorated by COR treated at 20 mg/kg. Further, the increased LPO was accompanied by the decrease in the levels of key antioxidant enzymes namely, SOD, CAT, GPx, GR and GST in the testis of aged rats when compared with those in young group. These enzymes are considered to be part of the body’s antioxidant defense systems. Furthermore, the non-enzymatic scavenging antioxidant system including the small molecular weight compounds such as reduced GSH, vitamin C and vitamin E also neutralize the free radicals. It is well documented that ageing-mediated oxidative stress conditions also effects the functional levels of non-enzymatic antioxidant defense system showing a decline from their normal levels (Cao et al. 2004; Liguori et al. 2018). In agreement with the published reports, in our study, we observed a significant decrease in the levels of GSH, vitamin C and vitamin E in aged rats when compared with young group. Treatment with COR (20 mg/kg) ameliorated the decreased levels of these contents indicating that COR regulated the functional imbalance of both enzymatic and non-enzymatic antioxidant defense systems and might enhance the testicular function in aged rats. It is likely that COR upregulated enzymatic antioxidant defense system, which in turn scavenge the generated free radicals and maintains non-enzymatic antioxidant contents.

Increasing amount of evidence suggests that ROS can induce apoptosis in germ cells and during ageing there is an increased oxidative stress-mediated apoptotic events in human testis (Jiang et al. 2014; Akhigbe and Ajayi 2020). Further, testicular oxidative damage and loss of reproductive function was also linked to caspase-mediated testicular apoptosis (Akhigbe and Ajayi 2020). Further, changes in lipid peroxidation and the expression of antioxidant enzymes with occurrence of apoptotic events has been observed in testicular ageing (Matzkin et al. 2016). In the present study, to understand the effect of COR on ageing-mediated apoptotic signalling we evaluated the pro- and anti-apoptotic protein expression in aged rat testis. Earlier reports indicated that several proteins control the process of apoptosis in ageing, including pro-apoptotic regulator Bax and anti-apoptotic regulator Bcl-2. Bcl-2 members promote cell survival while Bax promote cell death and their relative abundance in any cell may determine its fate (Chung and Ng 2006; Zhao et al. 2017). Further, studies have also indicated and increase in the levels of the ageing-related indicators such as p19, p21, and p53 in ageing testicles. Moreover, the activity of the tumour suppressor protein p53 is predominantly increased which has an association with cell cycle arrest triggering cellular senescence (Gambino et al. 2013). ROS over production may also facilitate sperm and testicular cells apoptosis by promoting the expression of P53 which in turn enhancing apoptosis signalling by increasing the expression of Bax or decreasing the expression of Bcl-2 (Xi et al. 2017; Zhao et al. 2017). In the present study, we found significant ageing-related changes in the levels of apoptosis regulatory proteins, namely, P53, Bcl-2, and Bax expression. The results demonstrated that the increased p53 and Bax protein expression and decreased Bcl-2 expression in aged rats were reversed following treatment with COR. Our results are in agreements with earlier reported data that an increased level of p53/Bax expression and a decrease in Bcl-2 levels in the skeletal muscle of aged rats (Chung and Ng 2006; Xi et al. 2017). However, further studies in *in vivo* ageing-mediated testicular impaired experimental models to understand the in-depth mechanisms of COR in in ameliorating male reproductive dysfunction caused by oxidative stress and ageing are quite essential.

## Conclusions

The present data suggests that COR treatment ameliorated H_2_O_2_-induced oxidative damage and alterations in spermatogenesis-related molecules in TM3 Leydig cells. Further, COR restored the antioxidant defense status in aged rats and ameliorated the alterations in apoptosis regulatory molecules in aged rats. Therefore, COR might be developed as a potential agent in protecting against ageing-associated oxidative stress-induced male infertility.
